# Analyzing Neuronal Mitochondria *in vivo* Using Fluorescent Reporters in Zebrafish

**DOI:** 10.3389/fcell.2018.00144

**Published:** 2018-10-25

**Authors:** Amrita Mandal, Katherine Pinter, Catherine M. Drerup

**Affiliations:** Unit on Neuronal Cell Biology, NICHD, National Institutes of Health, Bethesda, MD, United States

**Keywords:** mitochondria, neuron, dynein, kinesin, zebrafish, axonal transport

## Abstract

Despite their importance for cellular viability, the actual life history and properties of mitochondria in neurons are still unclear. These organelles are distributed throughout the entirety of the neuron and serve many functions, including: energy production (ATP), iron homeostasis and processing, calcium buffering, and metabolite production, as well as many other lesser known activities. Given their importance, understanding how these organelles are positioned and how their health and function is maintained is critical for many aspects of cell biology. This is best illustrated by the diverse disease literature which demonstrates that abnormal mitochondrial movement, localization, size, or function often correlates with neural pathology. In the following methods article, we will describe the techniques and tools we have optimized to directly visualize mitochondria and analyze mitochondrial lifetime, health, and function in neurons *in vivo* using fluorescent reporters in the zebrafish. The zebrafish system is ideal for *in vivo* studies of mitochondrial biology as: (1) neuronal circuits develop rapidly, within days; (2) it is genetically accessible; and (3) embryos and larvae are translucent allowing imaging in a completely intact vertebrate nervous system. Using these tools and techniques, the field is poised to answer questions of mitochondrial biology in the context of neuronal health and function in normal and disease states.

## Introduction

Mitochondria and the eukaryotic cells in which they reside are considered symbiotes. This now critical organelle is thought to have originally entered into existence as a bacteria that took up residence in another cell, likely a eukaryote. From that time until now, co-evolution of mitochondria and the cells of plants and animals have given rise to a situation in which each depends upon the other for survival. During this co-evolution, mitochondria have taken on many responsibilities in the cell. In addition to their well-known role in Adenosine Tri-Phosphate (ATP) synthesis, mitochondria function to buffer and store calcium, produce essential metabolites, synthesize signaling molecules, and regulate iron and iron processing. Despite these essential functions, we have only a minimal understanding of mitochondrial life history in cells. In particular, our knowledge of mitochondrial properties in neurons is specifically lacking. We will describe the tools developed by our lab and others that utilize zebrafish to study mitochondrial health, function, movement, and turnover *in vivo*, in order to understand mitochondrial life history in neurons.

### Mitochondrial structure and function

Mitochondria are double membrane-bound organelles. The canonical structure, starting from the outside, includes an outer membrane, inner membrane space, inner membrane and matrix. The curvature of the inner membrane creates the observed cristae. The most well-characterized function of mitochondria is producing ATP, the primary source of energy for cellular activities. This process occurs within the mitochondrial cristae. In neurons, the demand for ATP is incredibly high due to their high metabolic rate even at resting states. It is estimated that average activity in rodent gray matter, for example, uses ~30 μmol ATP/g/min (Attwell and Laughlin, 2001). Since an average rat brain is ~2 g, this means that every hour a rat brain is burning 3.6 mmol of ATP.

The generation of ATP in mitochondria occurs through the conversion of the pyruvate generated from glycolysis to acetyl CoA (the first step of the Kreb cycle; localized to the mitochondrial matrix). Acetyl CoA is then used to generate the carbon dioxide necessary to produce NADH/FADH_2_, the substrates of oxidative phosphorylation, i.e., ATP production. For this process to proceed, an oxidative potential must be maintained across the inner membrane. To create an oxidative potential, the inner membrane space is maintained at high hydrogen ion concentrations compared to the matrix, this creates a pH and charge gradient within mitochondria. Maintenance of mitochondrial matrix potential is tightly linked to mitochondrial health and several methods have been developed to monitor this aspect of mitochondrial biology. The most common method is the cationic dye tetramethylrhodamine ethyl ester (TMRE). This vital dye accumulates in the mitochondrial matrix due to its negative charge. Consequently, the fluorescence intensity of the mitochondrial TMRE is a readout of matrix potential and health. Though commonly used in cultured cells, the ability to measure matrix potential *in vivo* has, to date, been lacking. The maintenance of mitochondrial matrix potential is accomplished by a complex array of proteins intricately arranged in specific compartments within the organelle. Of particular importance are the respiratory chain complexes I-IV. These complexes maintain the proton and pH gradients across the inner membrane that are critical to ultimately power ATP synthase, generating ATP. Interestingly, a subset of the main components of the electron transport chain in mitochondria are encoded by the mitochondrial DNA, while the rest of the Complex I-IV components and the majority of the proteins that form and maintain the mitochondria are synthesized externally from nuclear DNA.

In addition to generating ATP, mitochondria serve as a calcium buffer and reservoir in cells. Calcium enters mitochondria through the largely unselective Voltage Dependent-Anion Channel (VDAC) (Gincel et al., 2001). Once in the inner membrane space, calcium is transported to the mitochondrial matrix by the mitochondrial calcium uniporter (MCU). For calcium release back to the cytoplasm, mitochondria utilize both sodium-dependent and independent calcium channels. Mitochondrial calcium levels are highly regulated and thought to rely on the cytoplasmic calcium concentration, in addition to other signals (Kirichok et al., 2004). Consequently, high calcium levels in the local microenvironment can result in rapid uptake of calcium into the mitochondrial matrix. In the mitochondria, calcium levels regulate mitochondrial functions as well as signaling molecules associated with cell death and cell survival (reviewed in Pivovarova and Andrews, 2010). Thus, calcium levels must be tightly regulated in mitochondria to ensure organelle function and cell viability are maintained.

Calcium regulation by mitochondria is especially critical in neurons. High cytosolic calcium levels have been linked to axonal and neural degeneration. This is likely due to the fact that mitochondria harbor cell death genes whose release regulates apoptosis. When calcium levels remain elevated in this organelle, mitochondria release these proteins and induce apoptosis, leading to loss of neural tissue. While high calcium levels on a prolonged basis lead to cell death, regulated elevation of calcium levels in mitochondria stimulates ATP synthesis (McCormack and Denton, 1989). Transient increases in cytoplasmic and consequently mitochondrial calcium levels commonly occur in neurons, particularly at synapses. Action potentials triggered by circuit activity lead to the activation of presynaptic calcium channels and calcium influx. Following calcium store release, this ion must be rapidly removed from the cytosol to regulate synaptic release. Both the endoplasmic reticulum (ER) and the mitochondria have been proposed to serve as intracellular stores that can rapidly buffer calcium after neuronal activity, though their relative contribution is still a source of active research and debate. It is likely that these organelles actually function in concert to regulate calcium ion levels as they are tightly coupled at regions of ER-contact sights shown to influence mitochondrial activity and signaling (Boehning et al., 2004; Cárdenas et al., 2010; Raturi et al., 2016). However, the role of mitochondria in calcium homeostasis has been difficult to address *in vivo*. We have optimized approaches to use genetically encoded calcium indicators (GECIs) to assay cytoplasmic and mitochondrial calcium levels *in vivo*. GECIs are commonly used to measure transient increases in intracellular calcium in neurons as an indicator of neuronal activity. These indicators typically consist of a calcium binding domain fused to one or two fluorescent proteins. The binding of calcium changes the fluorescence intensity of the signal. Common GECIs include GCaMP variants and GECOs. Particularly useful are the GECO color variants, including the red indicator R-GECO1, which has been used previously to study hair cell responses to mechanical stimulation (Maeda et al., 2014). The combination of calcium indicators with different spectral properties allows monitoring of whole cell calcium levels and subcellular compartments simultaneously as described below. As calcium serves to regulate neuronal activity, neuronal maintenance, and has a critical role in regulating mitochondrial movement, understanding the dynamics of mitochondrial-cytoplasmic calcium flux *in vivo* is of significant importance.

Finally, mitochondria can also act as signaling centers in cells. During the process of oxidative phosphorylation, mitochondria produce metabolites including NADH, FADH, succinate, reactive oxygen species (ROS), and many others. These molecules, sometimes thought of as byproducts, have been shown to induce cellular responses (Chandel et al., 1998; Sena and Chandel, 2012; Weinberg and Chandel, 2015; Weinberg et al., 2015). For instance, evidence in cancer cell lines has demonstrated that mitochondrial metabolites can in fact signal to regulate the growth and movement of these cells, making this organelle a target for cancer therapy (Wang et al., 2011; Weinberg and Chandel, 2015). In addition, work in neurons has demonstrated that mitochondrial positioning can regulate the localization of axon branching (Courchet et al., 2013; Spillane et al., 2013). While this could be attributed to the higher levels ATP in the microenvironment surrounding this organelle, it is entirely possible that the signaling molecules produced locally could regulate subcellular dynamics necessary for axon branching to initiate as well. With the incredible potential of mitochondrial metabolites to influence the local microenvironment, the maintenance and regulation of mitochondrial health and positioning within the neuron is of obvious importance. To date, it has been difficult to measure mitochondrial metabolite production *in vivo*. We have developed a protocol to use transient transgenic animals that express an indicator of chronic ROS production in neurons. This protein, Timer, is oxidation sensitive, switching its fluorescence spectrum based on oxidation state (Hernandez et al., 2013; Laker et al., 2014). With this new tool, we have the ability to assay chronic ROS in various neuronal compartments in normal states and with manipulation.

### Mitochondrial dynamics

Mitochondria are not static, but are rather quite dynamic organelles. One activity of particular importance for mitochondrial maintenance is the active interchange of mitochondrial components, known as mitochondrial dynamics. The term mitochondrial dynamics describes the fusion events that bring two mitochondria together as well as the fission events which produce two daughter mitochondria from a single parental organelle. Work on mitochondrial dynamics has shown a clear role for fusion in the maintenance of mitochondrial health. Studies in which mitochondrial fusion has been disrupted have shown that this leads to loss of mitochondrial DNA and subsequent mitophagy (Chen et al., 2005, 2010; Chen and Chan, 2009). Several mitochondrial proteins have been identified as necessary for mitochondrial fusion, including Mitofusin and OPA1 (Optic Atrophy 1; Alexander et al., 2000; Delettre et al., 2000; Zuchner et al., 2004). Loss of either protein results in highly fragmented mitochondria, degradation of these organelles, and subsequent neuronal cell death. The necessity of fusion is thought to be due to in its ability to replenish proteins and mitochondrial DNA quality and quantity, maintaining the organelle's health and function. Of similar importance is the process of mitochondrial fission. During fission, receptors on the mitochondrial outer membrane, such as Fis1 and Mff, recruit the dynamin-related protein Drp1 to mitochondria. Drp1 oligomerizes, resulting in a constricted point that then cleaves to generate two independent organelles. While the process of fission is clearly important for the maintenance of a healthy mitochondrial pool (Parone et al., 2008; Twig et al., 2008; Ban-Ishihara et al., 2013), the underlying mechanistic reasons for its necessity, particularly in neurons, are somewhat unknown. A subset of literature suggests that fission may be a way for the “unhealthy” parts of the organelle to be targeted for degradation. Evidence to support this includes the lower membrane potential observed in one of the two daughter mitochondria following division; however, the fate of the daughter mitochondria after fission is not clear. Therefore, the actual function of mitochondrial fission in the maintenance of a healthy mitochondrial pool in neurons is still an active area of investigation.

### Mitochondrial transport

A process intricately related to mitochondrial dynamics is mitochondrial transport. Active transport of this organelle occurs in all cell types, but neurons are perhaps most keenly sensitive to the precise regulation of this process due to their large size and high metabolic demand. Neurons, unlike many cell types, have large processes which can stretch up to a meter away from their cell bodies in humans, as is the case for a subset of axons that make up the sciatic nerve. This means that their axon length to cell body diameter ratio is roughly 100,000 to 1. In order to form and maintain this enormous structure, proteins and organelles must be actively transported through the cell body and processes using molecular motors and their microtubule tracks. These tracks are unipolar in axons, with their plus or fast-growing end situated toward axon terminals. The directionality of these tracks governs the direction of movement by microtubule-based motor proteins. Anterograde axonal transport (away from the cell body/toward microtubule plus ends) utilizes a superfamily of Kinesin motors proteins (Pilling et al., 2006). Conversely, retrograde axonal transport (cell body-directed/toward microtubule minus ends) is primarily accomplished by a single motor protein complex, Cytoplasmic Dynein (Schnapp and Reese, 1989). The mechanics and regulatory mechanisms that control how and when these motors attach to and move mitochondria through axons is tightly regulated and a subject of intense investigation.

Anterograde mitochondrial movement is largely accomplished by the Kinesin-1 molecular motor. To bind to mitochondria, Kinesin-1 elicits the help of adaptor proteins Miro (a Rho GTPase) and Milton (also known as Trak1/2). Originally discovered in Drosophila, these proteins are essential for movement of mitochondria from the neuronal cell body into axons via anterograde transport (Stowers et al., 2002; Glater et al., 2006; Russo et al., 2009). A key discovery in the regulation of mitochondrial movement came due to the study of the structure of Miro in particular. Miro is a small Rho GTPase that contains EF hand structural domains. These EF hand domains bind calcium which elicits a conformational change in the protein that changes the propensity for the Kinesin motor to interact with microtubules, which is necessary for processive mitochondrial movement (Wang and Schwarz, 2009). It is likely that Miro and Milton have a role in retrograde transport of mitochondria as well as loss of either effects transport in both directions. In support of this, elevated calcium levels stop all mitochondrial movement (Russo et al., 2009; van Spronsen et al., 2013). Therefore, an understanding of how direction of mitochondrial transport is actively controlled is still lacking.

A step toward understanding how mitochondria move in either the anterograde or retrograde direction came recently as a result of a forward genetic screen in zebrafish. This genetic approach identified the protein Actr10 as essential for mitochondrial attachment to the retrograde motor protein complex (Drerup et al., 2017). Actr10 is a member of the dynein accessory complex dynactin. It is situated in the so called pointed-end complex of dynactin, in an ideal place for cargo binding. Loss of this protein in zebrafish caused mitochondrial accumulation at microtubule plus ends due to failed mitochondrial retrograde transport (Drerup et al., 2017). Anterograde mitochondrial transport is not affected by loss of Actr10, further suggesting the importance of this protein specifically for regulation of retrograde mitochondrial movement. Below we describe the tools we have developed that allowed us to characterize the retrograde transport defect in this line. We also present the subsequent technologies we have adapted for use in this system which are allowing us to define the ultimate function of retrograde mitochondrial transport in neurons.

## Methodology and results

### Using zebrafish to discover the mechanisms and function of retrograde mitochondrial transport

Zebrafish larvae are an ideal system to identify and characterize the mechanisms that regulate axonal transport *in vivo*, as we and others have shown (Drerup and Nechiporuk, 2013, 2016; O'Donnell et al., 2013; Campbell et al., 2014; Paquet et al., 2014; Drerup et al., 2017). Embryos develop rapidly with primary neural circuits developed by 4 days post-fertilization (dpf). Zebrafish are also translucent through these stages, allowing visualization of cellular and sub-cellular phenomena *in vivo*. In addition, zebrafish are a genetically tractable system: they are amenable to forward and reverse genetic screens, transient and stable transgenic animals are easily generated, and a wealth of transgenic and mutant lines are available through community databases. Finally, zebrafish have well-characterized neural circuits for analysis of the functional implications of transport disruption. One well-characterized circuit is the afferent axons of the posterior lateral line (pLL) mechanosensory system.

**Figure 1:**
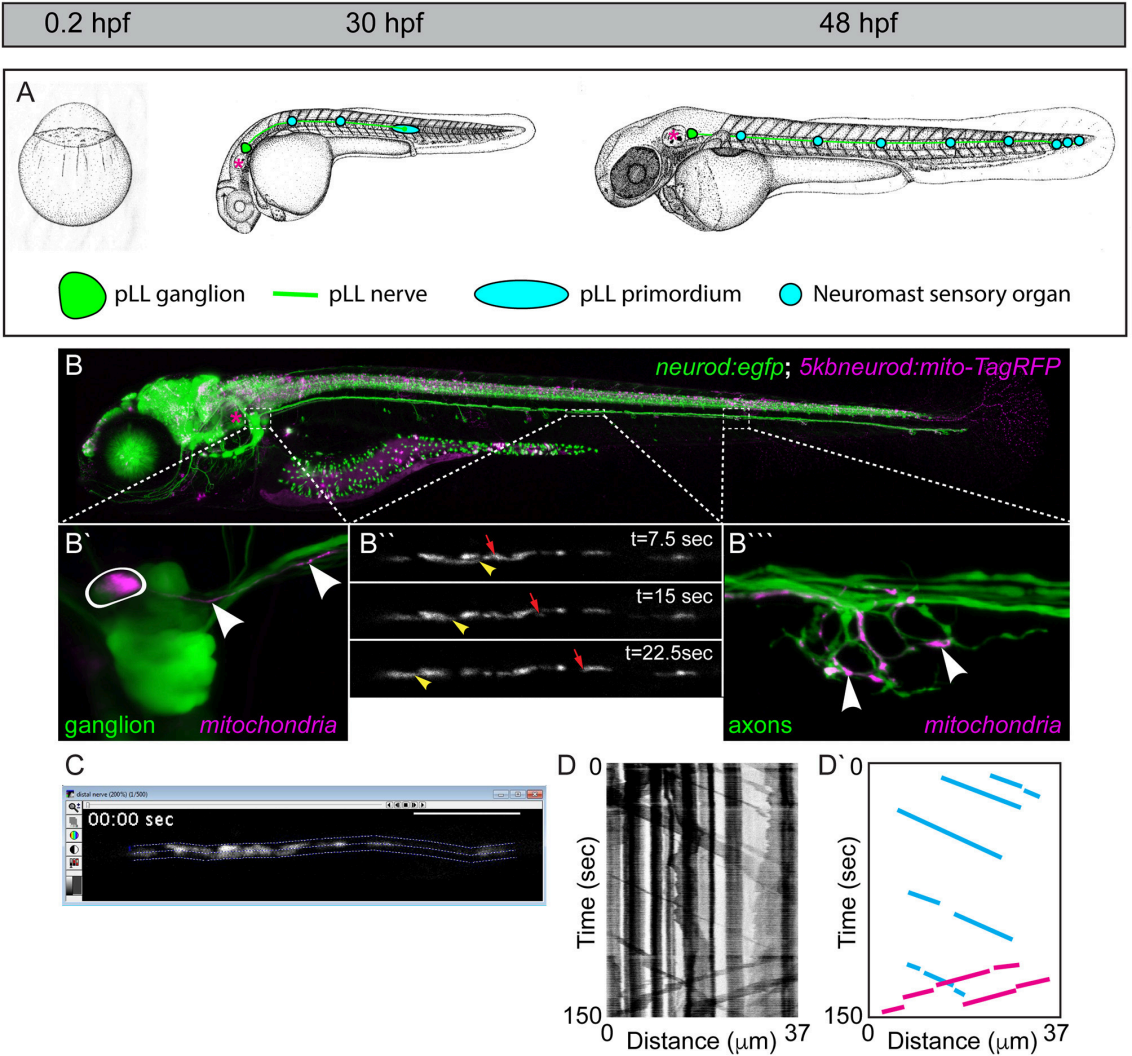
The zebrafish posterior lateral line (pLL): a circuit for addressing mitochondrial transport, health, and function. **(A)** The zebrafish develops rapidly from a single cell stage zygote to a hatched larvae by 48 h post-fertilization (hpf). During that time, the primary pLL also develops. Derived from a single ectodermal placode, the pLL primordium (blue oval) migrates from just behind the ear (asterisk) to the tip of the tail, completing its migration by 48 hpf. Along the way, the pLL primordium deposits approximately five nascent neuromasts which will develop into the sensory organs of the pLL. As it migrates, the pLL primordium guides the axons of the pLL nerve to the tail. The axons stretch from the pLL ganglion (green; contains pLL cell bodies) which remains behind the ear (asterisk) to the terminal cluster neuromasts in the tail in the longest of cases. Images modified from Kimmel et al. (1995). Permissions to use these images were obtained from the copyright holder, John Wiley & Sons, Inc. **(B)** By 5 days post-fertilization (dpf; bottom) the zebrafish larva is freely swimming and feeding. This image is of a live transgenic zebrafish larvae at 5 dpf which carries the stable *TgBAC(neurod:egfp)*^*nl*1^ transgene (hereafter referred to as *neurod:egfp*) that labels all pLL neurons with cytoplasmic green fluorescent protein (GFP). This larva is also a *5kbneurod:mito-TagRFP* transient transgenic, which expresses mitochondrially-localized TagRFP (shown in magenta) in the mitochondrial inner membrane space in neurons. Transient transgenics are chimeras, with only a subset of neurons expressing this construct. **(B**′**)** Transient expression of *mito-TagRFP* in a single pLL ganglion neuron (outlined) and the axon extending from it (arrowheads). **(B**″**)** Mosaic expression allows imaging of a mitochondrial movement in a single axon of the pLL nerve. Yellow arrowhead points to a mitochondria moving in the retrograde direction. Red arrow indicates an anterogradely moving mitochondria (see Movie 1). **(B**^‴^**)** This single axon forms a basket-like axon terminal that innervates half of the hair cells in this neuromast. Arrowheads point to individual mitochondria in the terminal innervating sensory cells which are not shown. **(C)** Screenshot of an imaging analysis session in MetaMorph. The dotted lines outline the axonal region analyzed. **(D)** Kymograph of this imaging session. Mitochondrial movement is represented by the sloped black lines. Vertical lines show pauses in movement. **(D**′**)** Schematic of this kymograph with representative anterograde movement bouts (blue lines) and retrograde movement bouts (pink) traced.

The pLL is a sensory system in aquatic vertebrates that allows the animal to detect movement in the water around them (Dijkgraaf, 1963). This system is composed of clusters of sensory hair cells situated in neuromasts that are distributed along the flanks and throughout the head. The hair cells themselves have apical protrusions that bend in response to water movement. The mechanical force due to this bending activates hair cells and ultimately leads to the release of glutamate at the synaptic contact they make with the pLL afferent axon terminals (Obholzer et al., 2008). For our purposes, the pLL axons are ideal for studies of both the molecular regulation of mitochondrial transport and the impact that disruption of this process has on the function of the circuit. The pLL forms in the first several days of development with initial axon extension completed by 2 dpf (days post-fertilization) and functional circuits by 4 dpf (Figure 1; Metcalfe, 1985; Metcalfe et al., 1985). The axons of the pLL nerve extend from the cell bodies of the pLL ganglion, located behind the ear, and to the tip of the tail in the longest cases. These axons are just under the skin and are largely planar, making visualization easy and reliable. Finally, unlike other sensory neurons, the axons of pLL neurons have stereotyped projections and well-defined axon terminals which allows rapid detection of any abnormalities in structure and function (Faucherre et al., 2009; Sarrazin et al., 2010; Dow et al., 2018). Because of the ease of visualization, mitochondrial transport can be imaged *in vivo* without removing neurons from their natural environment. In this way, neurons, myelinating glia, pre- and post-synaptic components, extracellular structural proteins and molecules, and growth factor support, as well as other more amorphous factors found in the surrounding tissue can be studied in an intact system. As neural circuit activity, growth factors, and myelination have all been proposed to effect mitochondrial transport (Chada and Hollenbeck, 2004; Kiryu-Seo et al., 2010; Ohno et al., 2011), the zebrafish pLL axons are highly suited for understanding the cellular mechanisms that regulate the movement of this organelle *in vivo*.

A critical question in the field of mitochondrial transport is the relevance of retrograde movement to mitochondrial health and neural circuit function. While anterograde mitochondrial transport is necessary to populate the axon and facilitates axon outgrowth (Morris and Hollenbeck, 1993; Han et al., 2016; Zhou et al., 2016), the precise function of retrograde mitochondrial transport is less clear. Evidence from cultured mammalian neurons has demonstrated that mitochondria with lower oxidative potential, thought to be a sign of failing health, are more likely to be transported in the retrograde direction (Miller and Sheetz, 2004; Lin et al., 2017). This work has been used to suggest that the main role of mitochondrial retrograde transport is to remove damaged organelles from the axon; however, there are conflicting reports that indicate that oxidative potential may not influence direction of mitochondrial movement (Suzuki et al., 2018). In addition to understanding the exact relevance of mitochondrial retrograde transport, there are several unexplored questions that are fundamental to axonal mitochondrial transport, health, lifetime, and function. First, how often do mitochondria move in axons? Second, does movement change with development? Third, does mitochondrial health and function differ between different regions of the cell? Finally, do mitochondria serve to regulate calcium levels differentially in subcellular compartments? Below, we will describe the tools we have developed to address these questions.

### Analyzing mitochondrial transport in the pLL

Two primary systems exist to analyze mitochondrial movement in axons *in vivo* in the zebrafish (O'Donnell et al., 2013; Paquet et al., 2014; Drerup and Nechiporuk, 2016; Drerup et al., 2017). Both use sensory axons and mark these organelles with fluorescent proteins to then monitor their active movement. The method developed independently by the Sagasti and Misgeld groups utilizes a GAL4:UAS system to drive expression of mitochondrial markers in axons of Rohon-Beard cells, a type of cutaneous sensory neuron in the early embryo (O'Donnell et al., 2013; Paquet et al., 2014). Because these axons arborize across the skin, mitochondrial localization and movement can be easily imaged in single axons *in vivo*. However, the precise synaptic targets of these cells are less clear and systems are not in place to monitor synaptic activity, complicating analyses of circuit structure and function. As an alternative system, we have optimized the pLL sensory system for interrogation of mitochondrial transport regulation and function in zebrafish axons. As described above, this system has stereotyped synapses and analysis of circuit function is routine (Zhang et al., 2016). Finally, several reagents, as outlined below, have been generated that allow targeting of mitochondria in single axons in the pLL nerve, allowing analysis of mitochondrial movement *in vivo*.

To visualize mitochondrial transport, we have optimized the system that we used previously to analyze lysosome and dynein movement in pLL axons (Drerup and Nechiporuk, 2013, 2016; Drerup et al., 2017). In this system, zebrafish zygotes can be injected with a plasmid encoding a mitochondrial targeting sequence derived from Cytochrome C Oxidase fused to an open reading frame encoding a fluorescent reporter. Expression is driven in neurons using a five kilobase portion of the *neurod* promotor (*5kbneurod*; Mo and Nicolson, 2011). As zebrafish embryos and larvae develop they exhibit mosaic expression of the reporter in pLL neurons (Figure 1B). At the stage of interest, the zebrafish are screened, and those with expression of the mitochondrial marker in one to two neurons of the pLL ganglion are selected for analysis. Individuals are then mounted on a coverslip in 1.5% low melt agarose and imaged at high magnification on a confocal microscope (e.g., a 63X, NA1.2 objective with a 100 × 100 μm field of view on an LSM800 microscope). To capture mitochondrial dynamics, imaging is done in a single *z*-plane, in a 30–100 μm length of axon, three to five times a second, as per Nyquist sampling requirements. The region imaged for analysis is chosen based on the ability to follow a single organelle through the region of interest, ensuring the visualized axon (~1 μm diameter) is kept in a single *z* plane (±0.44 μm; Figure 1C; Movie 1). Axonal transport of mitochondria can be imaged in various regions of the expressing pLL axon using this set up, with a consistent area chosen between related experiments. Typically, this imaging is done in the middle portion of the trunk, between neuromasts two and four (see Figure 1B), near the end of the yolk sac extension after 2 dpf. For imaging during axon extension, we image a region approximately two thirds of the distance between the neuronal cell body and growth cone of the extending axon. Mitochondrial transport distance, velocity, and direction can then be assayed directly from these image sequences (Drerup and Nechiporuk, 2016; Drerup et al., 2017).

Mitochondrial transport parameters are measured using kymograph analysis in MetaMorph (BioVision). The transport session is analyzed to ensure that individual organelles can be tracked through the length of the region of interest analyzed and the axon region to be analyzed is identified in the program (Figure 1C). Kymographs of the fluorescent signal are then generated using MetaMorph (Figure 1D). These line traces represent the organelles imaged with longer organelles generating thicker traces. Each individual movement bout, represented by a slanted line that can be followed from beginning to end, is traced (Figure 1D′). The distance and time of a single movement bout are identified as the change in X and Y, respectively, on the associated kymograph. The slope then translates to the velocity of movement. Finally, the number of mitochondria moving or stationary is manually counted. While kymographs can be used to estimate these values, we have found that manual assessment is more consistent.

**Figure 2:**
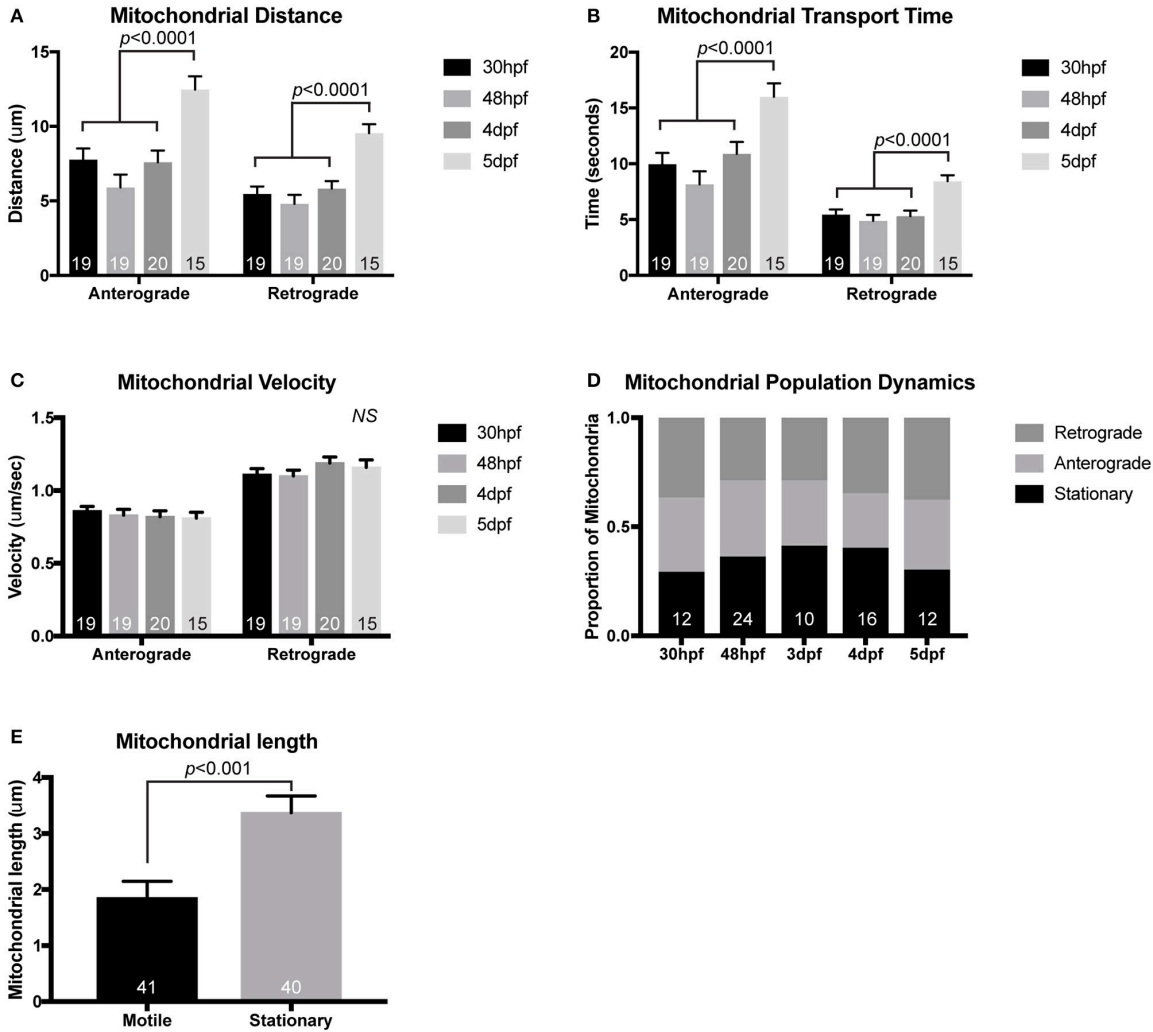
Mitochondrial transport during and after initial pLL development. While mitochondrial transport is fairly consistent during (30 hpf) and after initial axon extension (48 hpf), distance of mitochondrial movement in single bouts of transport **(A)** and the time that the mitochondria can move without stopping **(B)** is increased by 5 dpf (ANOVA with *post-hoc* contrasts). **(C)** Velocity of mitochondrial movement is constant at all ages analyzed. **(D)** Similarly, the proportion of mitochondria moving in either the anterograde or retrograde direction and the proportion that are stationary is not changed across developmental stages (ANOVA with *post-hoc* contrasts). **(E)** Stationary mitochondria are larger than those moving in either direction (ANOVA). *NS*, not significant. In **(A–D)**, the number of larvae analyzed is indicated on the graph. In **(E)**, the number of individual mitochondria analyzed from eight individual larvae is shown.

Using this general imaging scheme, we have analyzed mitochondrial transport parameters across development in zebrafish embryos and larvae. After injection of the mitochondrial reporter construct, embryos were raised to 30 hpf, 2, 4, or 5 dpf prior to mounting and imaging. These time-points were chosen specifically to match up with critical developmental stages in the pLL circuit. At 30 hpf, pLL axons are mid-extension and have active growth cone dynamics (Metcalfe, 1985). By 48 hpf, extension of the longest “pioneer” axons of the pLL is complete, but pLL axon terminals synapses are not yet formed with target hair cells (Metcalfe et al., 1985). By 4 dpf the pLL neural circuit is complete. At pLL axon terminals, there are synapses formed with hair cells and active neurotransmission can be observed between pLL axons and their hair cell targets (Zhang et al., 2018). Finally, at 5 dpf myelinating glia are present on the pLL nerve and have wrapped the axons, though compression of the myelin sheath is not yet complete (Monk et al., 2009). When we analyzed mitochondrial transport parameters across these developmental stages we found that the distance and time of anterograde and retrograde movement was maintained at a steady state from 30 hpf to 4 dpf. At 5 dpf there were slight but significant increases in both the time and distance of anterograde and retrograde movement bouts compared to other time-points (Figures 2A,B; ANOVA with *post-hoc* contrasts). At all developmental stages, the velocity of mitochondrial movement remained constant at ~0.83 μm/s and ~1.14 μm/s for anterograde and retrograde movement, respectively (Figure 2C). These values fall within the large window of reported mitochondrial axonal transport velocities among various systems (MacAskill and Kittler, 2010) and align closely with velocities observed *in vivo* in mouse sciatic nerve studies (Misgeld et al., 2007). The underlying reason for the enhanced bi-directional transport at 5 dpf is not clear but could rely on the establishment of stabilized microtubules, the addition of microtubule modifying proteins, or perhaps the expression of adaptor proteins necessary for long-distance transport.

When we focused our developmental analysis on the direction of mitochondrial movement, among all stages, we found no significant change in the proportion of mitochondria that were moving in the anterograde or retrograde direction. Additionally, during development, there was no change in the proportion of mitochondria that were stationary in these axons (Figure 2D; ANOVA with *post-hoc* contrasts). This is somewhat contradictory to literature analyzing mitochondrial transport in cultured neurons where days *in vitro* correlated with decreased mitochondrial movement (Kang et al., 2008). The precise reason for these differences is not entirely clear but could be due to a number of reasons. One possibility is the nature of the larval zebrafish. While the primary pLL is fully developed by 5 dpf, the fish continues to grow. This could mean that the axons are not in an entirely mature state by this time-point and do not express all of the docking proteins required to decrease mitochondrial movement (Kang et al., 2008). Another factor that could account for the consistent mitochondrial population dynamics in the pLL axons is the *in vivo* nature of this analysis. Previous work on mitochondrial transport frequency has largely been done on *in vitro* culture systems or in the sciatic nerve of a mouse after removal of surrounding tissues. It is possible that disruption of the neuron's *in vivo* environment alters intracellular transport dynamics, particularly over long periods of time. Future work on adult neurons in zebrafish could allow us to differentiate between these possibilities but technically this is not feasible in the short-term.

### Measuring mitochondrial lifetime in axon terminals

**Figure 3:**
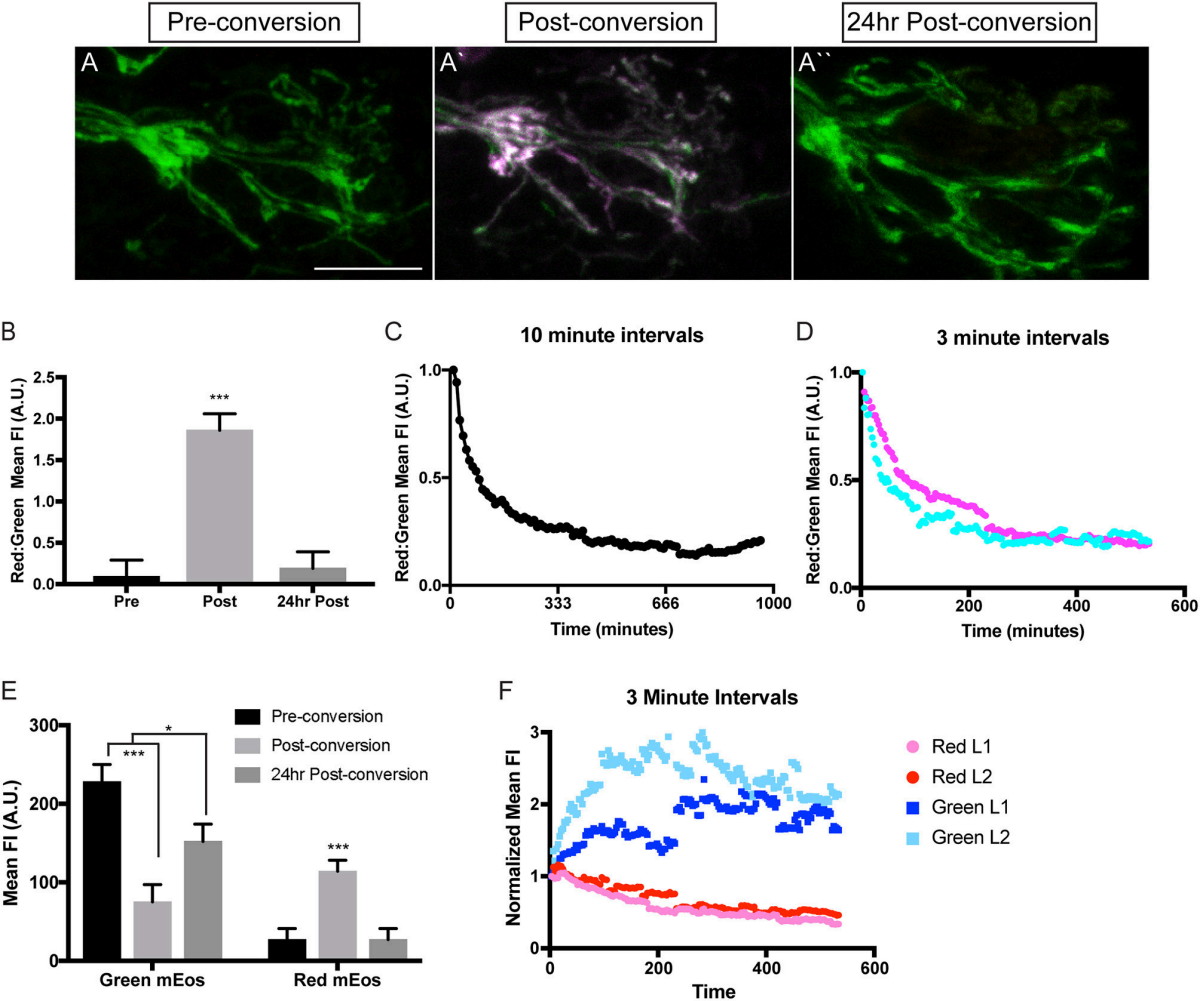
Analysis of mitochondrial lifetime in axons. **(A)** Expression of mitochondrially-localized mEos in the posterior lateral line of a 4 dpf *5kbneurod:mito-mEos* transgenic larvae. For our analysis we used *5kbneurod:mito-mEos* homozygotes to maximize mitochondria-localized mEos expression. This approach provides strong labeling of mitochondria in axon terminals. **(A**′**)** Illumination with 405 nm light converts mEos from green to red (shown in magenta). **(A**″**)** Twenty-four hours post-conversion, minimal converted (red) mEos remains in the axon terminal. **(B)** The relative converted to unconverted mito-mEos illustrates an almost complete loss of converted mitochondria from axon terminals by 24 h post-conversion (ANOVA with *post-hoc* contrasts; ***p < 0.0001; *n* = 15). **(C,D)** Time-lapse analysis of red to green *mito-mEos* in axon terminals after photoconversion shows a rapid turnover of mitochondria from the axon terminal, with most converted (red) mitochondria lost by 3 h post-conversion (*n* = 1 for **C**; *n* = 2 for **D**). **(E)** Quantification of the mean unconverted (green) and converted (red) mito-mEos in axon terminals shows both addition of new mitochondria and loss of converted mitochondria over a 24 h period (ANOVA with *post-hoc* contrasts; ***p < 0.0001; *p < 0.01; *n* = 15). **(F)** Time-lapse analysis of green and red mEos mean fluorescence intensity (normalized to post-conversion values) immediately after photoconversion in two larvae (L) demonstrates increased green and decreased red mEos over time. Timelapse data from two larvae normalized to post-conversion values. Scale bar = 10 μm.

Mitochondria can be long-lived organelles, undergoing dynamic rearrangement to sustain themselves and their viability. Work *in vitro* in cultured Drosophila and chick neurons as well as *in vivo* imaging of mitochondria in the exposed sciatic nerve of mice has shown that these organelles largely exist in two pools; a stationary pool comprised of longer mitochondria and a smaller, actively moving mitochondrial pool (Miller and Sheetz, 2004; Misgeld et al., 2007; Narayanareddy et al., 2014). This is recapitulated in the zebrafish pLL axons, where stationary mitochondria (~3.5 μm) are longer than motile mitochondria (<2 μm; Figure 2E). This demonstrates that there is consistency between experimental systems. Overall, analyses of mitochondrial transport dynamics have given us a substantial amount of information about the acute nature of mitochondrial movement. Unfortunately the majority of experiments analyzing stationary verses mobile mitochondrial pool have largely been done on time periods that span minutes. Therefore, little is known regarding how the properties of individual mitochondria within axons change over longer periods of time. In addition is not clear whether there are subtypes of mitochondria restricted within the cell body, axon, or axon terminal. To begin to address these questions, we have developed a stable transgenic line to express a photoconvertible protein, mEos (McKinney et al., 2009), in neuronal mitochondria [*Tg(5kbneurod:mito-mEos)*^*y*586^]. mEos was originally discovered in coral and is known for its ability to be stably photoconverted from green to red using 405 nm illumination. It is widely used in developmental biology to label independent cells to determine the origin of various tissues and is now used to follow the persistence of intracellular structures as well (Lam et al., 2015). We have utilized it similarly to track the localization and persistence of mitochondria in neuronal compartments. For this work, we engineered a transgenic fish in which mitochondrially targeted mEos (mito-mEos) is expressed in neurons using the *5kbneurod* promotor (Mo and Nicolson, 2011) and the mitochondrial targeting sequence from Cytochrome C Oxidase (Fang et al., 2012). We have previously used this signal sequence to transiently tag mitochondria with red fluorescent protein successfully (Drerup et al., 2017 and Figure 1). Analysis of our transgenic line indicates that mitochondria are efficiently tagged with mEos in this line and can be visualized in pLL axon terminals (Figure 3A).

In order to analyze mitochondrial lifetime in the pLL sensory axons, we examined larvae at 4 dpf, after pLL axons have formed stable synapses. For conversion and analysis, larvae were mounted in 0.8% low melt agarose for imaging with a 40X, NA1.0 dipping objective on a LSM800 confocal microscope (Zeiss). Pre-conversion, mitochondria were easily visualized with 488 nm excitation (green), indicating unconverted mEos. After short stimulation with 405 nm light, mitochondria were now visualized with 568 nm excitation (red), confirming mEos photoconversion (Figures 3A,B). To examine mitochondrial lifetime, imaging was done immediately after photoconversion and again 24 h later in axon terminals. Analysis of red to green fluorescence intensity revealed that immediately after photoconversion the mEos signal was dominated by red fluorescence (Figure 3B). In contrast, after 24 h there was an almost complete loss of the converted (red) mEos relative to green in axon terminals, indicating that there are high levels of mitochondrial turnover at this site (Figures 3A,B). Next, we analyzed the timeline of mitochondrial turnover using time-lapse imaging following photoconversion at 4 dpf. With both 10 and 3 min intervals, we found relative old (red) to new (green) mitochondria in axon terminals plateaued by ~3 h post-conversion (Figures 3C,D). This data implies a rapid turnover of mitochondria, within hours, in axon terminals.

Turnover could be due to either converted mitochondria moving out of the terminals, mitophagy in the axon terminal, or, perhaps, new (unconverted) mitochondria moving into the terminal, increasing the “green” signal. To differentiate between these possibilities, we analyzed the change in mean green and red fluorescence intensity immediately after photoconversion and compared it to the intensities after 24 h. We found that 24 h after photoconversion, there was an increase in green mEos labeled mitochondria, indicating ample addition of new organelles at the axon terminal. Simultaneously, there was a strong decrease in mean red fluorescence intensity, bringing the values back down to pre-conversion levels, representing loss of all if not almost all photoconverted organelles by 24 h post-conversion (Figure 3E). We then wanted to more precisely determine the temporal dynamics of mitochondrial gain and loss from axon terminals. For this analysis, we used timelapse imaging again to monitor the green and red mEos intensity at 3 min intervals immediately after the photoconversion described above. Strikingly, there was a sharp increase in green mEos within 3 h after photoconversion with a concomitant decrease in red fluorescence intensity (Figure 3F). This indicates both addition of new mitochondria and loss of old occurs within this time period and both contribute to the ratiometric changes shown in Figure 3. The lower fold change in the red channel in Figure 3F is likely due to incomplete photoconversion (see Figure 3A′) Together, our data support rapid turnover of mitochondria in pLL axon terminals, with addition of new organelles (green) and loss of photoconverted (red/old) organelles on the span of hours. This high level of mitochondrial loss from axon terminals was surprising as previous studies in cultured neurons demonstrated large stationary pools of mitochondria, anticipated to remain in place for extended periods of time (Kang et al., 2008). Our data argues instead that these organelles are very dynamic in the axon *in vivo*, with rapid exchange over a few hours. One still open question is the ultimate fate of the axonal mitochondria lost from axon terminals. Subsequent work using this line will address the relative contribution of mitophagy verses mitochondrial transport in the turnover of mitochondrial axon terminal populations. Additionally, we plan to explore the relative lifetime of mitochondria in other regions of the neurons, including the cell body, to identify any changes in mitochondrial population dynamics related to cellular compartment. Together, this work will shed light on the lifetime and turnover of mitochondria in neuronal compartments *in vivo*.

### Measuring mitochondrial health and productivity *in vivo*

**Figure 4:**
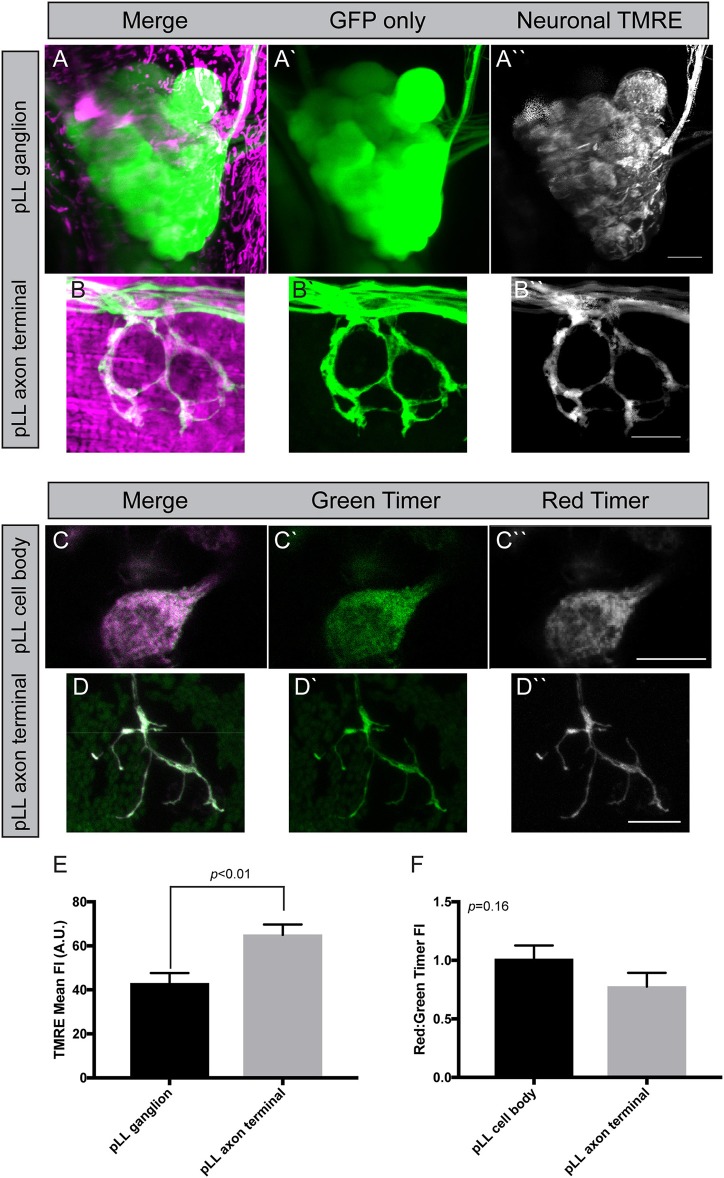
Measuring mitochondrial health in neurons. **(A,B)** Incubation of zebrafish larvae at 4 dpf with 25 μM TMRE results in strong labeling in pLL ganglia and axon terminals (shown in magenta). Cytoplasmic GFP marks the axons. Image subtraction in ImageJ allows analysis of TMRE labeling in neurons (identified by *neurod:egfp* transgene expression). **(C,D)** Transient transgenesis using a *5kbneurod:mito-Timer* construct allows imaging of the oxidation-sensitive protein Timer in individual pLL neurons and axon terminals in larval zebrafish at 4 dpf. Green Timer is native while red Timer (shown in magenta) is oxidized. **(E)** Quantification of TMRE fluorescence intensity shows elevated TMRE labeling in axon terminals compared to cell bodies, an indicator of higher matrix potential (ANOVA; *n* = 7 larvae). **(F)** The red:green Timer ratio is slightly, but not significantly reduced in axon terminals (ANOVA; *n* = 16 larvae). Scale bar = 10 μm.

Mitochondrial health is often assessed by analyzing the potential/pH across the inner membrane. Indeed, if the mitochondrial machinery that maintains this hydrogen gradient is not maintained, mitochondria fail to function normally and are subject to degradation. The most common way to analyze mitochondrial membrane potential is the vital dye TMRE (tetramethylrhodamine ethyl ester). This positively charged dye is highly attracted to the negative charge in the mitochondrial matrix, making it a marker of “healthy” mitochondria. We adapted a TMRE protocol used previously in the zebrafish pLL hair cells (Esterberg et al., 2014) for use in pLL sensory axons. For this experiment, we incubated zebrafish larvae at 4 dpf in 25 μM TMRE in embryo media with 0.1% DMSO (dimethyl sulfoxide) for an hour in the dark. Larvae were subsequently washed three times in embryo media prior to being mounted in 1.5% low melt agarose and imaged with a 63X, NA1.4 objective on a confocal microscope (Zeiss LSM800). For analysis, mitochondrial TMRE was measured in axon terminals and the pLL ganglion after subtraction of nonneural tissue using the ImageJ *Image Calculator* function. This revealed a consistent elevation in TMRE mean fluorescence in axon terminals compared to cell body mitochondria (Figures 4A,B,E). As TMRE recruitment is proportional to negative change in the matrix, this could mean that axon terminal mitochondria have a stronger oxidative gradient compared to those in the cell body. Alternatively, dye permeability or light scatter during imaging due to differences in tissue thickness could underlie this difference between pLL ganglion cell bodies and axon terminals. Therefore, we attempted to address the question of mitochodondrial health in another way, using the genetically encoded sensor, Timer (Laker et al., 2014).

Timer is a protein that fluoresces in the green spectrum when in its native conformation but switches to red upon oxidation (Laker et al., 2014). Therefore, the red (568 nm excitation) to green (488 nm excitation) ratio of Timer can be used to assess ROS production, an indicator of mitochondrial health. Excess ROS production is an indicator of oxidative stress and a failing mitochondrion prone to degradation. To express Timer in mitochondria, we used the mitochondrial targeting sequence described above to target it to the inner membrane space and expression was again driven using the minimal *5kbneurod* promotor sequence. Similar to what we described above, injection of the *5kbneurod:mito-Timer* construct resulted in mosaic expression of Timer in pLL neurons. Injected larvae were raised to 4 dpf, and we selected larvae with expression of Timer in a subset of pLL ganglion neurons. These larvae were mounted for imaging as described for TMRE. This analysis revealed no significant difference in the red to green Timer ratio in axon terminals verses the cell body; however, there was a trend toward lower red to green ratios, a potential indicator of lower oxidative stress, in axons terminals (Figures 4C,D,F; ANOVA *p* = 0.16). One important point to clarify, however, is that Timer does not just become oxidized based on elevated ROS in unhealthy mitochondria. ROS is a natural biproduct of oxidative phosphorylation. As mitochondrially localized Timer ages, it will naturally become oxidized as well, making the relative contribution of age verses increased oxidative stress difficult to fully distinguish without secondary methods, such as TMRE (Hernandez et al., 2013; Laker et al., 2014). Therefore, it is important to take the results of our mitochondrial turnover assays, TMRE staining, and mito-Timer analyses together. In combination, our work suggests that mitochondria in axon terminals are rapidly turned over, have a higher potential across their inner membrane, and perhaps have lower ROS production than those in the cell body. This, in turn, would allow axon terminal mitochondria to have increased ATP production relative to those in the cell body.

**Figure 5:**
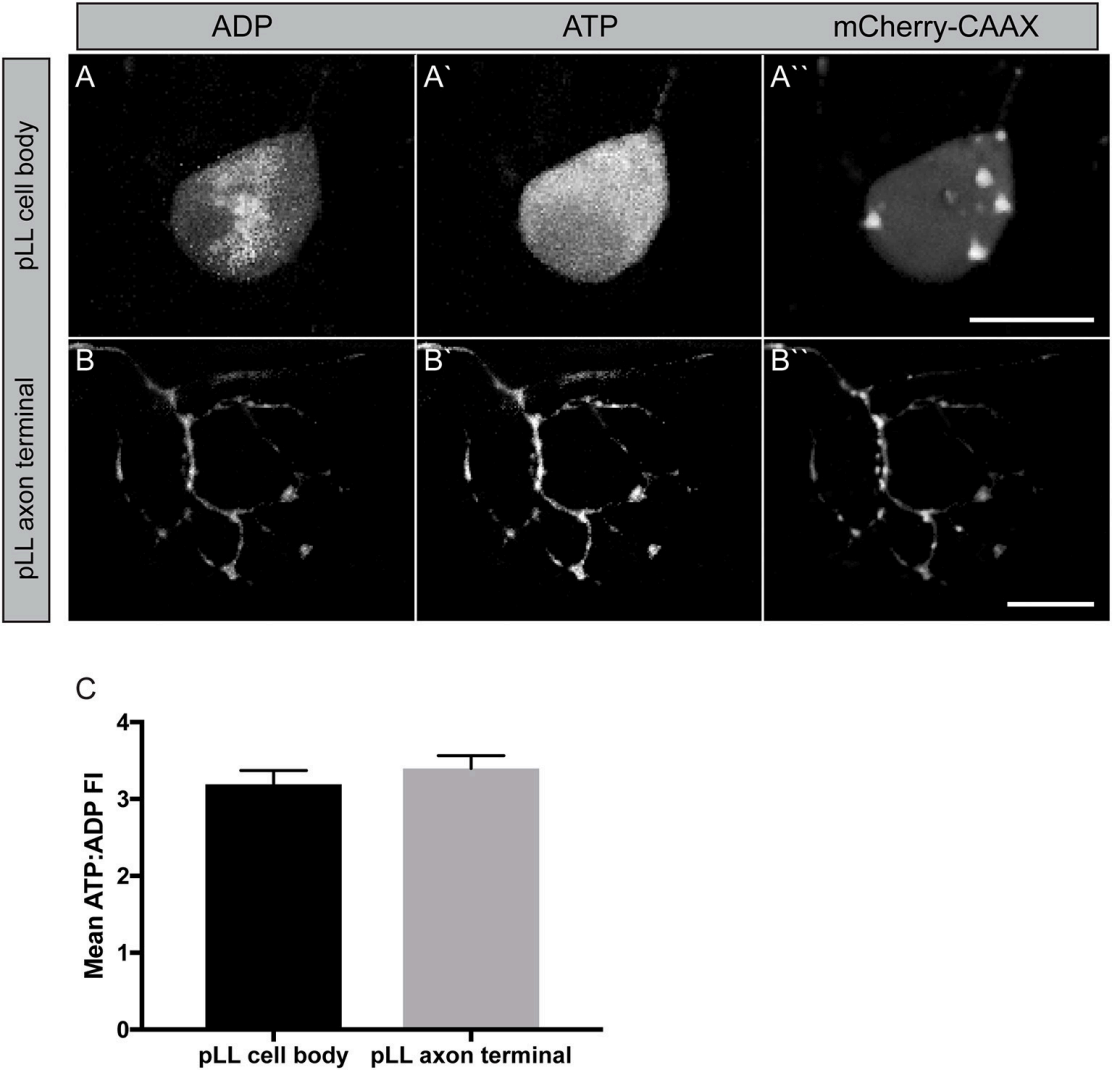
Measuring mitochondrial productivity *in vivo*. **(A,B)** Transient *5kbneurod:PercevalHRp2amCherry-CAAX* expression in a pLL neuron **(A)** and pLL axon terminal **(B)** allows imaging of ADP with 420 nm excitation **(A,B)** and ATP with 500 nm excitation **(A**′**,B**′**)**. mCherry expression used to identify the neuron. **(C)** Quantification of the ATP: ADP ratio showed similar levels of mitochondrial productivity in these regions (ANOVA; *n* = 21 larvae). Scale bar = 10 μm.

The next question we wanted to address was in regard to the actual productivity of mitochondria in the cell body and axon terminal of pLL sensory axons. For this, we used the ATP:ADP dual ratiometric sensor PercevalHR (Tantama et al., 2013). PercevalHR was developed as an improved version of the original Perceval: a chimeric protein, composed of the ATP-binding pocket of the bacterial protein GlnK1 and a mutated form of the yellow fluorescent protein mVenus (Berg et al., 2009). This protein can be competitively bound by both ADP and ATP with differential excitation peaks (420 vs. 500 nm, respectively) making it a ratiometric sensor. We made a DNA construct to transiently express PercevalHR in neurons, with a p2a cleavable peptide sequence linking it to mCherry-CAAX (membrane localized red fluorescent protein) to visualize the neuron. This *5kbneurod:PercevalHRp2amCherry-CAAX* construct, when injected into zebrafish zygotes, results in mosaic neuronal expression of the sensor in larval zebrafish. Quantification of the ATP:ADP ratio demonstrated consistent ATP:ADP ratios in axon terminals and the cell body of these neurons (Figure 5). Due to the somewhat variable nature of this transient transgenic approach, statistical comparisons between the cell body and axon terminals were challenging; however, as this sensor is ratiometric, our data imply that mitochondria in these distinct cellular compartments are adequately converting ADP to ATP. The generation of a stable transgenic line expressing this sensor in neurons, which is currently in progress, will allow comparative studies on mitochondrial productivity in various cellular compartments in the near future.

### Mitochondrial calcium buffering in neurons

The mitochondrial outer and inner membranes have calcium channels to rapidly take up this ion from the microenvironment surrounding this organelle. It has been proposed that this ability to locally modulate or buffer calcium could be of great importance to neurons, particularly at synapses (reviewed in Devine and Kittler, 2018). Synaptic activity requires active release of calcium from intracellular pools to facilitate neurotransmitter release. After activity, the calcium must then be sequestered to modulate the activity of the synapse. One method of calcium sequestration would be through mitochondrial uptake. In addition to calcium buffering, calcium entry into the mitochondria is also thought to stimulate mitochondrial productivity with elevated calcium levels increasing ATP synthesis; however, the mitochondrial calcium load must be properly regulated as prolonged elevation of mitochondrial calcium stimulates the release of pro-apoptotic factors and initiates cell death (reviewed in Strasser et al., 2000). Therefore, proper regulation of calcium by and in mitochondria is absolutely essential for mitochondrial and neuronal health.

**Figure 6:**
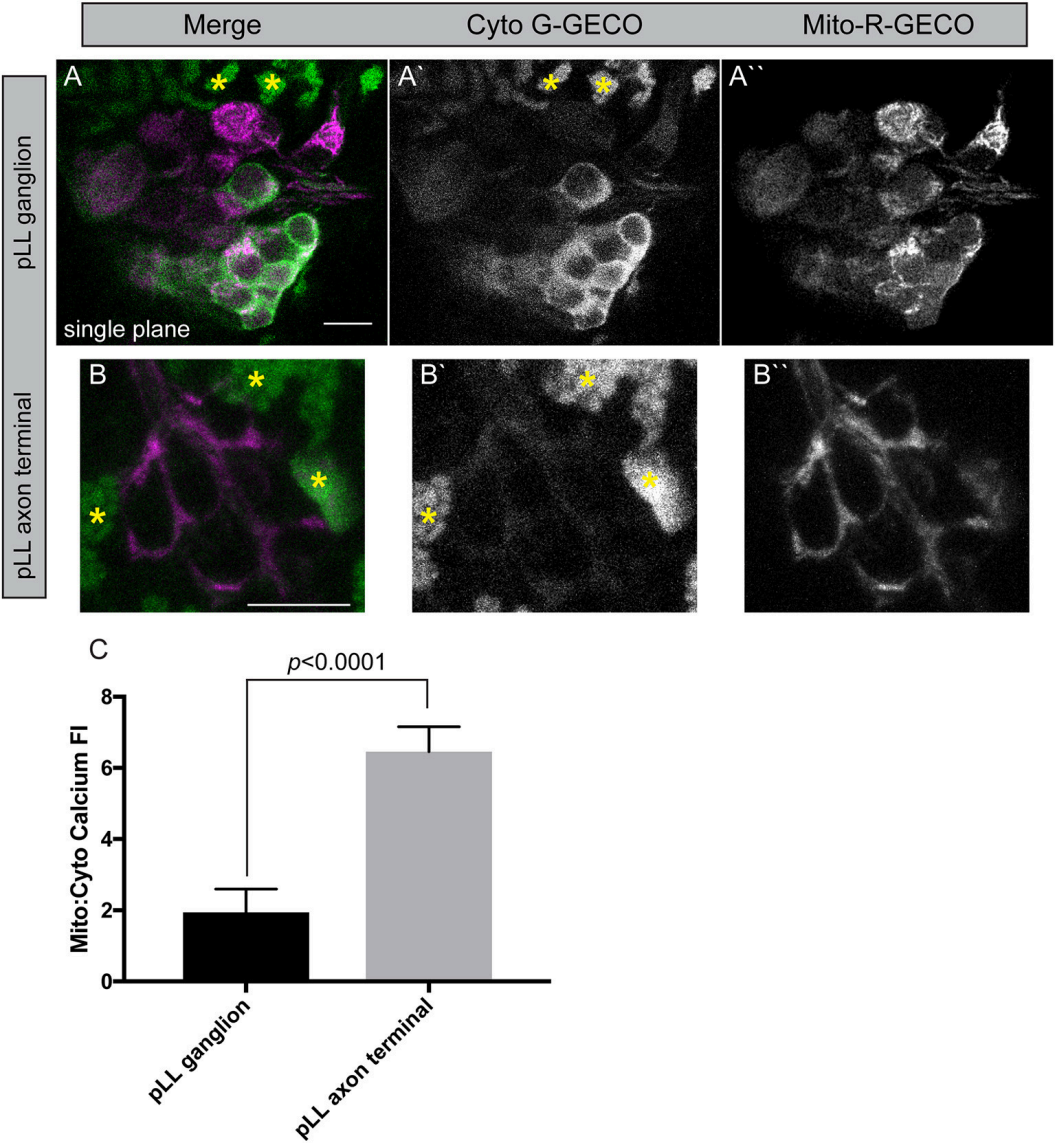
Mitochondrial calcium buffering in neurons. **(A,B)** Stable *5kbneurod:mito-R-GECO1* and *5kbneurod*:*G-GECO1* transgenics were crossed to generate trans-heterozygotes expressing cytoplasmic G-GECO (green) and mitochondrially localized R-GECO (magenta) in neurons. Asterisks indicate pigment cells that autofluorescence in the G-GECO channel. **(C)** Quantification of the mitochondrial to cytoplasmic GECO signals in the pLL ganglion and axon terminals demonstrates a strong bias toward mitochondrial calcium in axon terminals (ANOVA; *n* = 19 larvae). Scale bar = 10 μm.

To begin to study the relationship between cytoplasmic and mitochondrial calcium in neurons, we engineered two new transgenic lines. These lines are stable transgenics which express a green calcium indicator [G-GECO1; *Tg(5kbneurod:G-GECO)*^*nl*19^] in neuronal cytoplasm and a red calcium indicator [R-GECO1; *Tg(5kbneurod:mito-R-GECO)*^*nl*20^] in the mitochondrial inner membrane space (Zhao et al., 2011). Using confocal imaging as described for TMRE analysis, we can image chronic and acute changes in calcium ion abundance in and around mitochondria in these lines. This analysis revealed a striking difference between the neuronal cell body and axon terminals of the pLL sensory neurons. The mitochondria (red) to cytoplasmic (green) GECO ratio is increased in axon terminals compared to the cell body, indicating a higher calcium load in mitochondria compared to the surrounding environment in this compartment of the neuron (Figure 6). Together with the increased TMRE/lower Timer signal and rapid turnover, our data indicate that the axon terminal has an extremely dynamic population of mitochondria, that may be necessary to support synaptic activity and signal transduction.

In summary, our work has developed a collection of novel tools to interrogate the lifetime, health, productivity and function of mitochondria in neurons *in vivo* in the embryonic and larval zebrafish pLL sensory system. Our data to date illustrate the importance of considering all compartments when analyzing mitochondrial activity and transport, particularly the differences between organelles residing in the cell body verses those in the synaptically active regions of the axon terminal. Future work using these and other tools developed by the community will allow us to come to a better understanding of the symbiotic relationship between this former prokaryote and the eurkaryotic cell it calls home.

## Ethics statement

This study was carried out in accordance with the recommendations of NICHD Animal Care and Use Committee. The protocol (ASP 15-039) was approved by the NICHD ACUC.

## Author contributions

KP and AM contributed experimental design, data generation, and data analysis to this work. CD designed, conducted, and analyzed experiments and wrote the manuscript.

### Conflict of interest statement

The authors declare that the research was conducted in the absence of any commercial or financial relationships that could be construed as a potential conflict of interest.

## Supplementary material

Movie 1 Mitochondrial transport in a pLL axon. Mitochondria (labeled with TagRFP) are visualized moving in the anterograde (left to right) and retrograde (right to left) direction in a single pLL axon (see Figure 1). Scale bar = 10 μm.
